# Integrative Strategies for Preventing Nutritional Problems in the Development of Children in Brazil

**DOI:** 10.3389/fnut.2021.662817

**Published:** 2021-08-13

**Authors:** Mauro Fisberg, Lais Duarte Batista, Carlos Alberto Nogueira-de-Almeida, Flávia Mori Sarti, Maria Paula de Albuquerque, Regina Mara Fisberg

**Affiliations:** ^1^Feeding Difficulties Center, Institute PENSI (Pesquisa e Ensino em Saúde Infantil), Fundação Jose Luiz Setubal, Hospital Infantil Sabará (Sabara Children's Hospital), São Paulo, Brazil; ^2^Department of Nutrition, School of Public Health, University of São Paulo (USP), São Paulo, Brazil; ^3^Medical Department, Federal University of São Carlos (UFSCAR), São Carlos, Brazil; ^4^School of Arts, Sciences and Humanities, University of São Paulo (USP), São Paulo, Brazil; ^5^Section Physiology of Nutrition, Department of Physiology, Federal University of São Paulo (UNIFESP), São Paulo, Brazil

**Keywords:** nutrition, infant feeding, public policies, nutritional strategies, child development

## Abstract

Child healthcare has been a priority subject in several programs and public policies developed over the decades. However, initiatives implemented seem insufficient to overcome the challenges regarding the integral development and improvement of the nutritional status of children in Brazil. The initial developmental stages of a child include pregnancy, breastfeeding, and complementary feeding, which are determinants in future aspects of health and nutritional status. Therefore, the strategies addressing problems during these three periods of life have the potential to positively impact the promotion of healthy eating habits and food security throughout life. Developing countries with huge dimensions and vast inequalities, like Brazil, are marked by differences in regional, cultural, and social contexts that may hinder the implementation of programs and policies with a broad scope. Extensive operational and professional costs, in addition to time-consuming activities that are necessary to apply, monitor, and evaluate interventions may jeopardize the proper assessment of programs and policy goals, generating the inefficiency and waste of resources in the health system. Thus, programs and policies aimed at creating and modifying habits should consider an intersectoral action within local contexts, involving health professionals, universities, policy managers, and the community. Therefore, this article aimed to discuss the preliminary conception of an integrated approach of decentralized strategies to promote healthy eating habits and food security of children in Brazil.

## Introduction

Child healthcare has been a priority in several programs and public policies developed over recent decades, contributing to important changes in indicators of child health, and development. However, although these initiatives reinforce the prioritization of children in the broad scope of healthcare, they are still insufficient in dealing with challenges relevant to the integral development and improvement of the nutritional status of children from 0 to 60 months of age (1).

Throughout decades, diverse public policies and health programs have been implemented and extinguished in Brazil due to the lack of resources, absence of evidence regarding the effectiveness, or political disagreements (2). One of the main problems regarding the discontinuity of health programs or abrupt changes in the orientation of public health policies refers to a low adherence of the population to the recommendations of health professionals, considering the doubts raised, and confusion generated due to the instability of government actions (3).

The quality and frequency of the prenatal follow-up are still limited; the prevalence and duration of breastfeeding are still challenging, although there are several policies, programs, and recommendations available for guidance; similarly, feeding practices in first years of life are considerably far from guidelines. Since 1981, the National Breastfeeding Incentive Program has tried to induce a set of actions related to promotion, protection, and support of breastfeeding. In this context, over the years, the policies in Brazil have focused on the process of expansion of strategies that involve a wide range of actions developed in hospitals and primary healthcare, such as the Baby-Friendly Hospital Initiative (*Iniciativa Hospital Amigo da Criança*) and human milk banks (*Bancos de Leite Humano*). Another intervention performed in a primary care is the National Strategy for the Promotion of Breastfeeding and Healthy Complementary Food in the Primary Health Care System—*Estratégia Amamenta Alimenta Brasil* (EAAB), which seeks to promote, protect, and support breastfeeding until ≥2 years of age, being exclusively in the first 6 months of life and complemented with healthy foods from 6 months (1). However, even with such efforts over the last four decades, the prevalence of exclusive breastfeeding in Brazilian infants under 6 months of age is only 45.7% (4). Therefore, the evolution in important indicators of the health of a child still faces obstacles in the current context, including the high proportion of premature and low-birth-weight infants, one of the leading causes of preventable deaths in the first year of life (5). In addition, the low quality of food intake from complementary feeding has contributed to the increase of overweight and obesity among young children, concurrently with a high prevalence of nutritional deficiencies, a condition called “hidden hunger.” The current context of coronavirus disease 2019 pandemic has further intensified these problems, with important consequences for the quality of nutrition of children and child development. A recent study found that 59.4% of Brazilian households reported that they have experienced some level of food insecurity, as they did not have the adequate quantity and quality of food or as the concern that there would be a shortage of food compromises their choices (6).

The recent evidence shows that maternal feeding plays a leading role in the long-term health of a baby. After birth, exclusive breastfeeding up to 6 months of life is a decisive factor in the health of children and may impact adulthood. Finally, the period of food introduction may shape the quality of the diet, eating habits, and relationship with foods throughout life of children. This triad of periods involved in the initial aspects of child development—pregnancy, breastfeeding, and complementary feeding—is determinant of future aspects of the health and nutritional status of an individual. Therefore, the strategies addressing these important periods can positively impact the promotion of healthy eating habits and food security throughout life.

Thus, the aim of this study was to discuss the preliminary conception of a master plan to tackle the obstacles in the triad of periods decisive for child development—pregnancy, breastfeeding, and complementary feeding—through an integrated approach of decentralized strategies to promote healthy eating habits and food security of children in Brazil.

## Problems

### The Beginning of Everything: The Role of Adequate Nutrition From Pregnancy to Postpartum

The role of maternal nutrition influences the important aspects in child development. In this context, a maternal health status marked by nutritional deficiencies, poor diet quality, and presence of modifiable risk factors, such as smoking, may reflect on the health conditions from conception throughout life.

The availability and quality of access to health services and prenatal care are relevant for promoting healthy maternal nutrition, and its distribution throughout Brazil is still fragile. The adequacy and frequency of a prenatal follow-up comprise a challenge to overcome due to the low coverage by qualified professionals, resulting in barriers to regular access, and consequent prioritization in care, exclusively for pregnant women who are at a high risk.

Although there is a broad recognition of relevant themes involving child health and feeding in healthcare facilities, informational approaches are insufficient to solve the lack of maternal support. The rates of breastfeeding and early weaning, for example, still represent a challenge, even considering constant guidance in services and development of policies and programs for the promotion of breastfeeding since the decade of 1970s. In this sense, acknowledging the problem may be an insufficient stimulus for adopting practical actions in daily life, which may not be enough to promote changes in habits and improve indicators of interest, such as exclusivity and duration of breastfeeding.

### The Moment of Transition: Complementary Feeding in the Formation of Future Healthy Habits

Another fundamental moment in the formation of healthy eating habits among children comprises the process of introduction of complementary feeding, which should occur after 6 months of life. It is also a period of intense palate training that intensifies with the addition of new consistencies, colors, and flavors, and, simultaneously, it is a moment of a higher risk for exposure to inadequate foods with low nutritional value.

The practices of complementary feeding in Brazil are often marked by early introduction and high intake of inappropriate foods, especially nutritionally deficient items with a high content of added sugar, sweets, biscuits, and calories. Most children receive milk of cows prematurely, in replacement or complementarily to breast milk, being consumed by ~80% of children between 12 and 60 months of age. This results in excessive protein intake, which tends to reduce the consumption of other non-dairy foods and may cause critical nutritional deficiencies including iron, vitamin A, and fiber, among others (7).

Numerous factors influence the quality of diet supplied in the first months of life, thus, guidance on the nutritional value, and healthy food choices may be insufficient to tackle the problem. Issues, such as the access and availability of quality food in the neighborhood, permissive parental style, active family participation in planning and encouragement of appropriate eating practices, and early exposure to electronic equipment, are challenges that need to be recognized and addressed for the development of multifaceted strategies that incorporate the complexity of these factors in promoting healthy eating during childhood.

### The Abyss of Communication: When Recommendations of Health Professionals Are Not Heard

Historically, vertical educational strategies are the cornerstone of policies for encouragement of breastfeeding, promotion of adequate infant feeding, and follow-up of pregnant and post-partum women. However, these strategies have been unsuccessful in converting knowledge into practices in daily life of the target audience. These educational “microinterventions” are ineffective and usually present high direct and indirect costs due to the need for time-consuming activities of health professionals, particularly in primary healthcare, like the Family Health Strategy (FHS) in Brazil, designed to provide primary healthcare for individuals during regular household visits through interdisciplinary teams.

It is important to acknowledge that food consumption represents more than a simple biological act to fulfill the need for survival, being also a social practice. Therefore, the strategies aimed to promote adequate eating habits should consider the complexity of determinants involved in food choices, going beyond the concept of healthy food or its role in the health of an individual, which comprises an approach exclusively or predominantly based on the physiological context of food.

The household environment, the lifestyle choices of parents, and the intrafamilial relationships play prominent roles in infant feeding, generally determining the nutritional quality and quantity of food, food preferences, time, and the interval between meals and eating practices and behaviors of a child. Therefore, the cultural, economic, and psychosocial backgrounds of the family are relevant to drive the formation of positive experiences with an impact on infant feeding and, potentially, throughout life.

## Potential Solutions and Alternatives

### Fragmenting to Enlarge When Local Glance Becomes Part of Global Solution

Developing countries with huge dimensions and vast inequalities, like Brazil, are marked by differences in regional, cultural, and social contexts that may hinder the implementation of programs and policies with a broad scope. Extensive operational and professional costs, in addition to time-consuming activities that are necessary to apply, monitor, and evaluate the interventions, may jeopardize the proper assessment of programs and policy goals, generating inefficiency, and waste of resources in health system.

The decentralization of planning, implementation, and evaluation of initiatives may be an alternative to overcome these obstacles. The primary strategy within the decentralized approach would rely on creating small-scale “sentinel centers” directed for tangible interventions, allowing to generate knowledge and scientific evidence using a limited number of local resources. These sentinel centers would comprise the center for action of the pediatrician, linked to other health services, universities, or municipalities, in different regions of the country.

In addition to the partnership with universities distributed in different regions, empowerment and stimulation of active participation of policy managers, along with the engagement of the community, are necessary for the relevant and effective performance of these monitoring centers. A strategy that may integrate this approach with the potential for success is the initiative “Mayor Friend of the Child,” from the Abrinq Foundation (*Fundação Abrinq* or Foundation of the Brazilian Association of Toys Manufacturers), which is responsible for engaging policy managers in the implementation of actions and public policies aimed to promote and ensure rights of children and adolescents in Brazilian municipalities^1^. Therefore, a pilot program could explore the potential of municipalities already committed and dedicated to actions of interest in the subject toward the implementation of sentinel centers based on public-private partnerships. This collaborative process can contribute to the actions and activities of programs already implemented in the health system, like EAAB, which could enhance the expected results from these strategies and improve the health indicators of the population.

Regional centers located in different parts of the country would comprise a network of facilities adopting immediate and site-specific preventive interventions adapted to local context, including hundreds of health professionals in a single project to monitor food and nutritional status of children. A short-term follow-up measure would encompass centers throughout the country that collect and organize the data into a central unit. The actions would be remotely monitored and assessed to comprise the results comparing diverse local interventions in a practical way. Targeted strategies could be developed in each of the sentinel centers, contributing to identify successful strategies, according to the following proposals.

#### Strategy 1: Antenatal Childcare in Health Services

Childcare is the task for pediatricians who focus on monitoring the life of children in the primary healthcare. Considering recent recommendations on the early start of intervention for promoting the health of children, encompassing the period before conception (8), called preconception childcare, the pediatrician would participate in the counseling process of the couple who intends to have children (9), to ensure that the conception occurs more healthily. However, considering that it is not easy to engage individuals in preconception childcare, it would be essential to involve parents in the follow-up by a pediatrician at least during the gestational period using protocols of antenatal childcare (10).

Recommendations regarding the frequency of consultations and approaches in antenatal childcare include attendance of a pediatrician in consultations with the pregnant mother, preferably accompanied by the father, at least once every trimester of pregnancy. The expanded follow-up proposal should include approaches focusing on the health of a child, from monitoring the fetal growth, and preparing for immunizations to guidance on maternal nutrition, including the strategies for prevention of obesity and malnutrition and supplementation with micronutrients (11). The encouragement of breastfeeding would be one of the main focuses of the intervention, as it would be possible to prepare the family through training for the father and mother, focusing on the importance of breastfeeding and on strategies for it to be successful.

The proposal in the first strategy would be the implementation of antenatal childcare programs in a sample of primary healthcare facilities in different parts of the country (12), through a pilot project in which pediatricians would make three visits with pregnant women, one every quarter. The measurement of health outcomes like prevalence and duration of exclusive breastfeeding and adherence to immunization programs would be performed throughout a minimum period of 1 year in order to evaluate the effectiveness of the intervention.

#### Strategy 2: Continuous Training in Complementary Feeding for Professionals of the FHS

Health professionals become promoters of healthy eating when they can translate technical concepts into practical and accessible language among the community they assist (13). The Brazilian FHS provides an opportunity for dissemination of healthy lifestyles throughout local communities, considering its focus on the improvement of basic health practices including prevention and early detection of health problems through household visits and recurrent interactions with families (14). The conviviality allows the family members to spread changes in habits, e.g., good practices in food preparation, hygiene, and cleaning.

Thus, the proposal of the second strategy is based on a process of permanent education on complementary feeding for FHS professionals, adopting a hybrid format of conceptual activities and training in action with the following aims:

- Expansion of knowledge on complementary feeding;
- Improvement in communication skills of professionals regarding complementary feeding, using the tool developed in the project^2^. Experiences that Feed (in Portuguese, *Experiências que Alimentam*);
- Improvement of food and nutrition education practices; and
- Training health professionals to conduct meaningful conversations in health education.
- Development of a digital model of the tool aforementioned.

#### Strategy 3: Reduction of Inequalities in Nutritional Quality of Diets With Focus on the Family

Considering that healthy and unhealthy behavioral patterns are developed and maintained within the family, it serves as a basic social context and reference point for the development of behavioral characteristics. The utilization of the Internet through mobile, mainly the smartphones, has been a viable alternative to online cable connections in Brazil.

Approximately, 1,127 million Brazilians have access to mobile Internet and instant messaging applications like “WhatsApp,” adopted by 93% of the population^3^. In order to build on the potential of information and communication technologies for the dissemination of knowledge, the proposal in the third strategy is based on the expansion of face-to-face interventions through continuous training based on technological channels. The actions developed should include:

- Adaptation of mobile technologies to send messages on healthy habits, considering cultural aspects of each region and availability of regional foods;
- Policies to encourage food consumption directed to protect health, including subsidies and social support for regional foods (15);
- Guidance from community leaders toward family engagement through messages about healthy eating, e.g., salt, sugar, and fat reduction in food items available for the local population; and
- Applying the principles in the Dietary Guidelines for the Brazilian population (16) to promote healthy eating habits, such as encouraging culinary practices in the family environment and recommending the consumption of natural or minimally processed foods, such as fruits, vegetables, grains, and pulses.

#### Strategy 4: Social Participation and Centrality of Individuals for Assertive Communication of Health Professionals

The social context plays a significant role in the choice of strategies adopted in the feeding of children. Food consumption practices are originated from the experiences constructed from sociocultural conditions and scientific and popular knowledge of each period. The social network and the community bond are also determinants in the formation of childcare practices, especially concerning breastfeeding, and infant feeding.

Often, professional guidance and follow-up are replaced by the advice of other family members who underwent the same experience, like mothers, or grandmothers. It is important to emphasize that these situations should not be excluding alternatives, but rather should be complementary.

Working jointly with the community, including community leaders and community health workers, might be a strategy that encourages the protagonism of individuals and community recognition of health promotion spaces. Some initiatives to expand the role of community for health promotion may include:

- Creation of spaces for monitoring of child health and development in an articulated network of health professionals, including an opportunity for socialization between mothers and pregnant women, clarification of doubts, and guidance in groups from community experiences;
- Community radio or podcast projects as space for listening and speaking with community participation, turning into an information tool shared by health professionals and community members as active guests and participants; and
- Use of technologies for recording and dissemination of contents produced, e.g., radio, that may be used later in other moments for training sessions and dissemination of information in waiting rooms of healthcare facilities. Community health workers can be important actors in bringing community and strategies closer together, acting in capturing and encouraging participation and in disseminating material within the community itself. Materials can be used, for example, during home visits as a “product of the community itself,” a communication mechanism produced “along with” the people and not only “for” the people.

The estimation of costs related to the operationalization of the activities implemented in each sentinel center would allow the selection of cost-effective strategies in terms of the social value per resources invested and projection of conditions required for expansion of coverage and dissemination of activities to other locations (*scaling-up*), mainly through the incorporation of social participation in the evaluation processes (17).

The assessment of direct healthcare costs would be based on the measurement of resources required for operationalization of each activity, followed by the pricing of each resource unit employed, through the six stages as follows (18):

1. Identification of procedures performed during each activity of the sentinel center, through the application of a semi-structured questionnaire to interview health professionals involved in its execution;
2. Identification of human resources, equipment, and materials employed for each procedure performed;
3. Estimation of units of time dedicated by human resources and equipment or units of other inputs spent for each service comprising each procedure performed;
4. Investigation of monetary values of resources involved in each procedure performed (salaries per hour of human resources, price and duration of equipment, and unit prices of other inputs required);
5. Calculation of direct costs of each procedure performed through the multiplication of units of each resource by its monetary value; and
6. Estimation of total direct costs of each activity implemented in the sentinel center by summing up the direct costs of each procedure involved in its execution.

Finally, using the information on health outcomes obtained from each activity performed, it would be possible to estimate the cost-effectiveness ratios of each strategy proposed, allowing the comparison of actions implemented in specific local contexts to select the best alternatives for dissemination in health policies and programs at the municipal level in the different regions of the country. Furthermore, the adoption of an incremental approach in the redirection of actions with lower impacts on the health of children may avoid traumatic ruptures in public health activities, generating greater consistency, continuity, and reliability in health policies and programs (13).

## From Planning to Implementation: Ways to Foster Prevention During Childhood in Brazil

The manuscript presented the preliminary conception of a master plan to tackle deficiencies in the triad of periods decisive for child development (i.e., pregnancy, breastfeeding, and complementary feeding) through a series of decentralized strategies that comprise a national framework toward the promotion of healthy eating habits and food security of children in Brazil, a developing country with large territory marked by substantial socioeconomic inequalities.

The first stage in the implementation of the strategies in the country should be based on the operationalization of a pilot study in the five regions of the country, namely, North, Northeast, South, Southeast, and Middle-West. The pilot study would be designed to encompass the strategies previously described into a set of “sentinel centers” selected for the analysis of feasibility, costs, and health outcomes that may be obtained in the initiative prior to the full implementation of the master plan.

The idea of the pilot study would be to perform a small-scale preliminary evaluation of the requirements, strengths, and potential frailties of the master plan, in order to incorporate the improvements to the initiative design prior to investment for its execution throughout the country. The pilot study would be conducted through collaboration among at least five major public universities in Brazil (minimum of one university from each region), coordinated by the Brazilian Ministry of Health in cooperation with the State and Municipal Secretaries of Health responsible for healthcare provision within the area selected for operationalization of the “sentinel centers.”

The “sentinel centers” in the pilot study would be established on priority areas of the five regions that would be selected based on the following criteria:

1. Analysis of socioeconomic, demographic, and health indicators referring to child health and food security;
2. Availability of infrastructure, material, and human resources within the structure of the public sector directed to primary healthcare;
3. Engagement and consent to participation in the study from the community and the stakeholders from the public and private sector at a local level.

In order to allow the comparison of results obtained by the “sentinel centers” established in the pilot study, we proposed that there should be at least 10 “sentinel centers” (two for each region) that would be implemented in Brazilian municipalities with comparable characteristics across regions (i.e., five small-scale low-income municipalities with the scarcity of infrastructure, material, and human resources, marked by poor performance in outcomes of the child health, and five medium-scale to medium-income municipalities with minimum infrastructure, material, and human resources, marked by intermediate performance in outcomes of the child health).

The operationalization of the strategies at the local level would be executed by local stakeholders with the support of state-level resources and initial coordination of the Brazilian Ministry of Health. The “sentinel centers” established in the pilot study would be assessed by multidisciplinary teams of researchers from the universities involved throughout the first year of implementation. At the end of each 4-month cycle of the pilot study, the results of the strategies proposed would be assessed and compared across “sentinel centers” and regions, in order to propose changes that may improve health outcomes obtained in the initiative.

Following the pilot study, the second stage of the implementation refers to continuous dissemination of “sentinel centers” to other Brazilian municipalities that will be selected according to the priority using the criteria previously proposed in the pilot study. The third and final stages of implementation would be based on the recruitment of other municipalities wishing to engage in the initiative in the subsequent periods, based on the dissemination of the results in local workshops performed by policymakers, street-level bureaucracy, health professionals, and university researchers, following a structure similar to the annual meeting of the National Council of State Secretaries of Management (*Conselho Nacional de Secretários de Estado da Administração*)^4^.

Staggered adhesion of Brazilian municipalities would allow to maintain the assessment of impacts of the initiative on child health, based on the statistical analyses of the data obtained throughout the process of implementation, using quasi-experimental design approaches and qualitative analysis of comparative case studies. Thus, eventual failures and setbacks could be addressed by evaluation of problems identified, followed by a redesign of the intervention at the local level, and dissemination of the experience to other “sentinel centers” through the annual meeting. The succession of action monitoring/evaluation redesign in the “sentinel centers” would be continuous to allow a permanent adaptation of the initiative to the local circumstances and to the community needs, potentially establishing a cycle that allows adaptation, and evolution of the public policy.

## Conclusion

Why challenges experienced for almost a century in the country remain on the political agenda due to the absence of adequate and permanent solutions? The change in health behaviors depends on several individuals, social, and environmental factors, thus, the programs and policies aimed at the creation and modification of habits should consider an intersectoral action within local contexts, involving health professionals, universities, policy managers, and the community.

## Data Availability Statement

The original contributions presented in the study are included in the article/supplementary material, further inquiries can be directed to the corresponding authors.

## Author Contributions

MF, CN-d-A, MPA, RMF, and FMS contributed to the conception and design of the study. LDB wrote the first draft of the manuscript. All authors wrote sections of the manuscript and contributed to manuscript revision, read, and approved the submitted version.

## Conflict of Interest

The authors declare that the research was conducted in the absence of any commercial or financial relationships that could be construed as a potential conflict of interest.

## Publisher's Note

All claims expressed in this article are solely those of the authors and do not necessarily represent those of their affiliated organizations, or those of the publisher, the editors and the reviewers. Any product that may be evaluated in this article, or claim that may be made by its manufacturer, is not guaranteed or endorsed by the publisher.

## Footnotes

^1^Fundação Abrinq. Programa Prefeito Amigo da Criança. [Online] *Fundação Abrinq*. Available at: https://www.fadc.org.br/o-que-fazemos/programa-prefeito-amigo-da-crianca (accessed October 19, 2020).

^2^Project directed to promote the dialogue between family members and health professionals about complementary feeding, based on the support and development of facilitation materials through collaborative construction, theory of change, and design thinking, for 24 months. Available at: Centro de Recuperação e Educação Nutricional. Relatório Final do projeto “Experiências que alimentam”. CREN—Centro de Recuperação e Educação Nutricional, 2019.

^3^Statista. Internet usage in Brazil: statistics & facts. In: Internet: demographics & use. Statista Research Department; 2020. Available at: https://www.statista.com/topics/2045/internet-usage-in-brazil/ (accessed January 26, 2020).

^4^ https://gestaoeplanejamento.com/pt/instituicoes/consad
